# Alcohol consumption and mortality in patients undergoing coronary artery bypass graft (CABG)-a register-based cohort study

**DOI:** 10.1186/s12872-016-0403-3

**Published:** 2016-11-11

**Authors:** Mads Phillip Kofoed Grabas, Steen Møller Hansen, Christian Torp-Pedersen, Henrik Bøggild, Line Rosenkilde Ullits, Ulrik Deding, Berit Jamie Nielsen, Per Føge Jensen, Charlotte Overgaard

**Affiliations:** 1 Department of Health Science and Technology, Public Health and Epidemiology Group, Aalborg University, Niels Jernes Vej 14, 9220 Aalborg Øst, Denmark; 2 Department of Clinical Epidemiology, Aalborg University Hospital, Sdr. Skovvej 15, DK-9000 Aalborg, Denmark; 3 Department of Anaesthesiology, Næstved Hospital, Ringstedgade 61, DK-4700 Næstved, Denmark

**Keywords:** CABG, Coronary artery bypass graft, Alcohol consumption, Mortality, Cox regression models

## Abstract

**Background:**

Previous studies have shown that compared with abstinence and heavy drinking, moderate alcohol consumption is associated with a reduced risk of mortality among the general population and patients with heart failure and myocardial infarction. We examined the association between alcohol consumption and mortality in coronary artery bypass graft (CABG) patients.

**Method:**

We studied 1,919 first-time CABG patients using data on alcohol consumption and mortality obtained from Danish national registers from March 2006 to October 2011. Alcohol consumption was divided into the following groups: abstainers (0 units/week), moderate consumers (1–14 units/week), moderate-heavy drinkers (15–21 units/week) and heavy drinkers (>21 units/week). Hazard ratios (HR) of all-cause mortality were calculated using Cox proportional hazard regression analysis.

**Results:**

The median follow-up was 2.2 years [IQR 2.0]. There were 112 deaths, of which 96 (86 %) were classified as cardiovascular. Adjustments for age and sex showed no increased risk of all-cause mortality for the abstainers (HR 1.61, 95 % CI, 1.00–2.58) and moderate-heavy drinkers (HR 1.40, 95 % CI, 0.73–2.67) compared with moderate consumers. However, heavy drinkers had a high risk of all-cause mortality compared with moderate consumers (HR 2.44, 95 % CI, 1.47–4.04). A full adjustment showed no increase in mortality for the abstainers (HR 1.59, 95 % CI, 0.98–2.57) and moderate-heavy drinkers (HR 1.68, 95 % CI, 0.86–3.29), while heavy drinkers were associated with an increased mortality rate (HR 1.88, 95 % CI, 1.10–3.21). There was no increased risk of 30-day mortality for the abstainers (HR 0.74, 95 % CI, 0.23–2.32), moderate-heavy drinkers (HR 0.36, 95 % CI, 0.07–1.93) and heavy drinkers (HR 2.20, 95 % CI, 0.65–7.36).

**Conclusion:**

There was no increased risk of mortality for abstainers (0 units/week) or moderate-heavy drinkers (15–21 units/week) following a CABG. Only heavy drinking (>21 units/week) were significantly associated with an increased mortality rate. These results suggest that only heavy drinking present a risk factor among CABG patients.

## Background

Alcohol consumption is associated with both harmful and beneficial health effects. Alcohol consumption increases the risk of death caused by oral cancer, cirrhosis and alcoholism [1], and heavy drinking increases the risk of all-cause mortality [2, 3]. In contrast, a beneficial effect of moderate alcohol consumption has been generally associated with reduced mortality, specifically for patients suffering from heart failure and acute myocardial infarction (AMI) [2–8].

Thus, while moderate alcohol consumption has beneficial effects in general, it is less certain how alcohol consumption influences patients with established coronary heart disease (CHD), particularly when surgical revascularization in the form of coronary artery bypass graft (CABG) [9] is needed. In terms of mortality, CHD is of considerable importance [9–11], and in cases of significant left main coronary artery stenosis, multi-vessel coronary disease or severe angina pectoris, CABG is recommended, which increases the blood supply to the heart, relieves pain and prolongs life [9].

In addition to established CHD, CABG patients often have several co-morbidities [12, 13] and high mortality rates. Consequently, it is important to determine whether strict recommendations regarding alcohol consumption also apply to this high-risk patient group.

A few studies have examined the influence of alcohol consumption on composite outcomes, including mortality, in CABG patients [14, 15]. In general, these studies have shown no harmful effects of alcohol consumption. As moderate alcohol consumption decreases the mortality risk in the general population, it is possible that moderate alcohol consumption may have beneficial effects or at least does not affect the risk of mortality in a high-risk population of CABG patients. Stroke, AMI, chronic obstructive pulmonary disease (COPD), liver disease, heart failure, renal failure, atrial fibrillation (AF), diabetes mellitus and hypertension have previously been identified as potential confounders in studies of mortality among CABG patients [12, 15]. Other potential risk factors are increased body mass index (BMI) [16–19], smoking [2, 17, 20], low household income [17], and low educational level [17]. However, previous studies of alcohol consumption and mortality in CABG patients [14, 15] were not able to account for the previously mentioned potential confounders, and furthermore, these studies had composite outcomes, including mortality. A more comprehensive investigation is needed to determine the influence of alcohol consumption on mortality in CABG patients.

The objective of this study was to examine the association between alcohol consumption and mortality among CABG patients, while considering a wide range of severe co-morbidities and other potential confounders.

## Methods

### Design and data sources

A nationwide register-based cohort study was conducted. The study included patients who underwent first-time CABG surgery between March 2006 and October 2011. We obtained data on alcohol consumption, mortality and covariates from seven administrative registers. From birth or at the time of immigration, all residents in Denmark are assigned a unique personal identification number (CPR-number), which enables linkage between national Danish registries [21].

The Danish National Patient Register, which contains information on all patients in Danish hospitals and diagnoses according to the International Classification of Diseases version 10 (ICD-10) [22], was one of the registers used. The Danish Anesthesia Database [23, 24] contains information on alcohol consumption, smoking status and BMI. This information is systematically registered by an anaesthesiologist with the aid of a standardized questionnaire prior to surgery; the questionnaire is filled in by the anaesthesiologist based on the information provided by the patient. The Danish Register of Causes of Death contains information on all-cause mortality and causes of death based on death certificates completed by a physician, according to the ICD-10 [25]. The Danish National Prescription Registry contains information on all redeemed prescriptions in Denmark, according to the Anatomical Therapeutic Chemical classification (ATC) [26], and the Income Statistics Register contains information on income and taxes for Danish residents [27]. The Population’s Education Register contains information on completed education levels; it is approved by the Danish Ministry of Education [28].

### Study population

All surgical procedures were registered according to the Nordic Medico-Statistical Committee (NOMESCO) classification of surgical procedures, which is used in Nordic countries [29]. The Danish National Patient Register was used to identify first-time CABG patients. Patients with the following surgical procedures were included (with related codes): connection to the coronary artery from the internal mammary artery (FNA), connection to the coronary artery from the gastroepiploic artery (FNB), aortocoronary venous bypass (FNC), aortocoronary bypass using a prosthetic graft (FND) (*n* = 1), coronary bypass using free arterial graft (FNE), coronary thromboendarterectomy (FNF) and repair of coronary artery (FNH). Patients were excluded if they also underwent other surgical procedures simultaneous, such as closure of coronary fistula (FNJ), repair of anomalous origin of a coronary artery (FNK), right ventricle and pulmonary valve (FJ), mitral valve (FK), aortic valve (FM), transplantation of heart or heart and lung (FQ), arrhythmias and disturbances of impulse propagation (FP), pulmonary artery with branches (FB) and thoracic and thoracoabdominal aorta, excluding malformations (FC). Patients were also excluded if they were diagnosed with cancer (ICD-10 C00-C97) prior to baseline.

### Exposure measure

The CABG operation dates, which were obtained from the Danish National Patient Register, were used as the date of entry. Patients were excluded if they not met the inclusion criteria in the study population section described above or did not have data on alcohol consumption registered. The alcohol consumption measurement date was obtained from the Danish Anesthesia Database, and only patients for whom alcohol consumption was measured less than three days before the operation date were included in the final study population. Alcohol consumption was divided into four groups: 0 units/week (abstainers), 1–14 units/week (moderate consumers), 15–21 units/week (moderate-heavy drinkers), >21 units/week (heavy drinkers). One unit of beer, wine or liquor contains approximately the same amount of alcohol, and alcohol consumption is often considered in units rather than in grams or millilitres of alcohol [30–32]. One standard unit of alcohol contain 12 g of alcohol [33], and moderate consumption is regarded as 1–2 units of alcohol per day [7].

### Outcomes measured

Information on mortality was obtained from the Danish Register of Causes of Death. Patients were followed until death or the end of follow-up, which was December 31, 2011 (censoring), whichever was first. In addition to all-cause mortality, we also examined cardiovascular mortality, which was followed up in the same manner, except that death from causes other than cardiovascular mortality was censured at the date of death. ICD-10 I00-I99 was used to define cardiovascular mortality. All-cause mortality also included cardiovascular death, and 30-day mortality was defined as patients dying within 30 days after the CABG operation date.

### Covariates

Stroke, liver disease, heart failure, renal failure, COPD, AMI, AF, hypertension and diabetes mellitus [12, 15] were included as potential confounders. Apart from hypertension and diabetes mellitus, diagnoses registered in the Danish National Patient Registry were used to determine whether the patients suffered from one or more of these diseases prior to the CABG operation. Redeemed prescriptions were used as a proxy for hypertension and diabetes mellitus prior to baseline [26]. Patients suffering from hypertension were identified by combination treatment with at least two classes of antihypertensive drugs, as previously described elsewhere [34]. If the patients claimed prescriptions for glucose-lowering medications (ATC A10) prior to baseline, then they were considered to have diabetes mellitus.

Information on BMI and smoking status was obtained from the Danish Anesthesia Database. BMI was divided into three groups: underweight (<18.5) or normal weight (18.5–24.9), overweight (25–29.9) and obese (>30). The grouping follows the BMI definition from the World Health Organization (WHO): <18.5 is underweight, 18.5–24.9 is normal weight, 25–29.9 is overweight and >30 is obese [35]. Smoking status was registered as never smoked, previous smoker or current smoker. Household income in Danish kroner (DKK) was divided into quartiles: quartile 1 (<218,122), quartile 2 (218,123–286,685), quartile 3 (286,686–418,426), and quartile 4 (>418,426). Education levels were obtained from the Danish Education Registers [28]. According to the United Nations Educational, Scientific and Cultural Organization’s (UNESCO) guidelines for classifying education (the International Standard Classification of Education 2011), the levels for highest completed education at baseline were classified as follows: basic school, high school education, vocational education, short or medium higher education and long higher education [36].

### Ethics

This study was approved by the Danish Data Protection Agency (Ref.GEH-2014-014). All data were linked and stored in computers held by Statistics Denmark and made available with encrypted CPR-numbers to ensure that no individuals were identified. In agreement with the Act on Processing of Personal Data, only aggregated statistical analysis and results are published [37, 38]. Written informed consent or ethical approval is not required for register-based studies in Denmark [37, 38].

### Statistical analysis

Analyses of variance (ANOVA) for continuous variables and Chi-squared (*x* ^2^) tests for categorical variables were used to test for between-group differences in weekly alcohol consumption. Survival analysis was performed, with Cox proportional hazard models. Hazard ratios (HR) and 95 % confidence interval (95 % CI) were estimated. Model 1 in the Cox regression was adjusted for age and sex, and the full adjustment in model 2 further included smoking status, BMI, household income, educational level, CABG after 2008 (time), hypertension, diabetes mellitus, COPD, AMI, AF, heart failure, liver disease, stroke and renal failure. *P*-values < 0.05 were considered to be statistically significant. Survival was estimated by the Kaplan-Meier method, and group differences were assessed with the log-rank test to examine the between-group differences. The analyses were performed with multivariate imputations using chained equations (MICE) on missing covariates (education level, smoking status and BMI) [39], as they increase the size of the study population and are typically more efficient than complete cases analyses [40, 41]. Thus, the main results are presented with the imputed data. For sensitivity analysis, the analyses were also conducted on the population with complete data. Tests for the interaction between alcohol consumption and covariates were conducted, and none were found. Data management was performed using SAS software, version 9.4 (SAS institute Inc. Cary, North Carolina, USA). Statistical analysis was performed using the R statistical software package, version 3.2.2 (R Development Core Team).

## Result

**Fig. 1:**
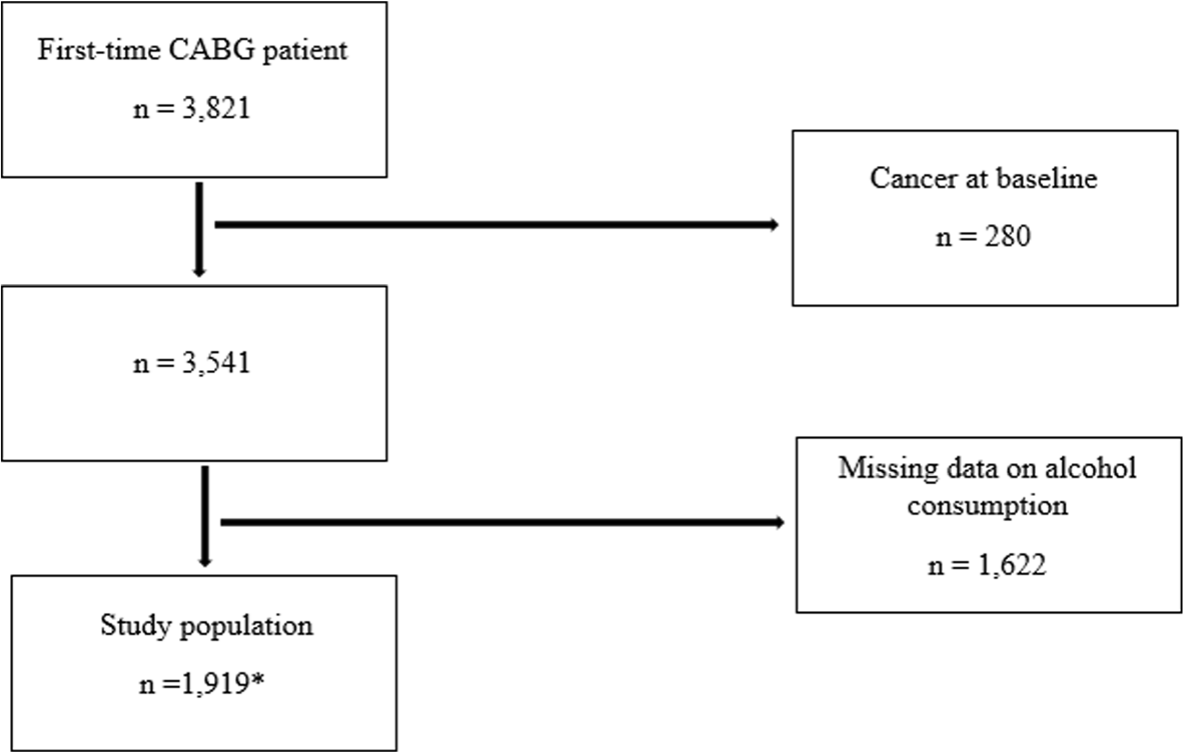
A flow chart of the exclusion process for the study population. CABG patients were identified in the Danish National Patient Register and from information in the Danish Anesthesia Database (3,821) between March 2006 and October 2011. Missing values for the following covariates were included due to the application of MICE: *99 patients were included with missing smoking status data, 54 patients were included with missing educational level data, and 2 patients were included with missing BMI data

In total, 3,821 first-time CABG patients were identified; however, 280 patients were excluded due to having cancer at baseline, and 1,622 patients were excluded due to missing alcohol consumption data. The final sample size was 1,919 (Fig. 1).

Baseline and demographic characteristics stratified by alcohol consumption are reported in Table 1. The patients had a median age of 67, and 83.4 % were males. The median follow-up was 2.2 years [IQR 2.0], (range 0–5.8 years). Of the 1,919 patients, 554 (28.8 %) reported alcohol consumption of 0 units/week, 783 (40.8 %) reported 1–14 units/week, 296 (15.4 %) reported 15–21 units/week, and 286 (14.9 %) reported >21 units/week. Of the study population of 1,919 patients, 99 had missing smoking status data, 54 had missing educational level data, and two patients had missing BMI data.

**Table 1:** Baseline characteristics of CABG patients by weekly alcohol consumption

Characteristics	Level	0 units (*n* = 554)	1–14 units (*n* = 783)	15–21 units (*n* = 296)	>21 units (*n* = 286)	Total (*n* = 1,919)	*P*-value
Sex	Male	394 (71.1)	647 (82.6)	291 (98.3)	267 (93.4)	1599 (83.3)	<0.0001
Age	median [IQR]	66.9 [13.1]	68.1 [13.1]	65.3 [10.6]	66.2 [11.8]	67.0 [12.5]	0.0039
Smoking status	Never smoked	158 (30.2)	186 (24.4)	58 (20.5)	36 (14.3)	438 (24.1)	
	Former smoker	255 (48.8)	443 (58.1)	147 (51.9)	145 (57.5)	990 (54.4)	
	Smoking	110 (21.0)	133 (17.5)	78 (27.6)	71 (28.2)	392 (21.5)	< 0.0001
	Missing	31	21	13	34	99	
BMI	Underweight or normal weight	149 (26.9)	280 (35.8)	86 (29.1)	82 (28.8)	597 (31.1)	
	Overweight	247 (44.7)	349 (44.6)	139 (47.0)	137 (48.1)	872 (45.5)	
	Obese	157 (28.4)	154 (19.7)	71 (24.0)	66 (23.2)	448 (23.4)	0.0019
	Missing^a^	.	.	.	.	< 3	
Household income^b^	Quartile 1	142 (25.6)	155 (19.8)	41 (13.9)	52 (18.2)	390 (20.3)	
	Quartile 2	118 (21.3)	140 (17.9)	44 (14.9)	60 (21.0)	362 (18.9)	
	Quartile 3	141 (25.5)	178 (22.7)	74 (25.0)	54 (18.9)	447 (23.3)	
	Quartile 4	153 (27.6)	310 (39.6)	137 (46.3)	120 (42.0)	720 (37.5)	< 0.0001
Educational level	Basic school	258 (49.0)	289 (37.6)	80 (27.6)	85 (30.5)	712 (38.2)	
	High school education	14 (2.7)	14 (1.8)	10 (3.4)	5 (1.8)	43 (2.3)	
	Vocational education	190 (36.1)	330 (42.9)	136 (46.9)	118 (42.3)	774 (41.5)	
	Short or medium higher education	47 (8.9)	96 (12.5)	40 (13.8)	56 (20.1)	239 (12.8)	
	Long higher education	18 (3.4)	40 (5.2)	24 (8.3)	15 (5.4)	97 (5.2)	< 0.0001
	Missing	27	14	6	7	54	
CABG after 2008		387 (69.9)	534 (68.2)	244 (82.4)	144 (50.3)	1309 (68.2)	< 0.0001
Hypertension		319 (57.6)	433 (55.3)	149 (50.3)	174 (60.8)	1075 (56.0)	0.0632
Diabetes		147 (26.5)	129 (16.5)	56 (18.9)	51 (17.8)	383 (20.0)	< 0.0001
COPD		33 (6.0)	42 (5.4)	17 (5.7)	16 (5.6)	108 (5.6)	0.9737
Heart failure		391 (70.6)	590 (75.4)	231 (78.0)	201 (70.3)	1413 (73.6)	0.0371
Liver disease		10 (1.8)	9 (1.1)	10 (3.4)	< 3 (< 0.9)	< 32 (<1.7)	0.0172
Stroke		62 (11.2)	68 (8.7)	31 (10.5)	31 (10.8)	192 (10.0)	0.4444
AMI		208 (37.5)	268 (34.2)	119 (40.2)	106 (37.1)	701 (36.5)	0.2845
Renal failure		26 (4.7)	21 (2.7)	6 (2.0)	10 (3.5)	63 (3.3)	0.1180
AF		43 (7.8)	56 (7.2)	15 (5.1)	34 (11.9)	148 (7.7)	0.0164

*BMI* Body mass index, *COPD* chronic obstructive pulmonary disease, *AMI* acute myocardial infarct, *AF* atrial fibrillation, *CABG after 2008* number of patients who underwent CABG surgery after 2008 (time)

^a^< 3 observations in some rows

^b^Household income is reported in Danish Kroner (DKK)

Compared to the other alcohol groups, abstainers (0 units/week) were characterized by a high proportion of obesity (28.4 %), diabetes mellitus (26.6 %), and more had basic school education (49.0 %) as their highest educational level. Moderate consumers (1–14 units/week) had a higher proportion of former smoking (58.1 %) and underweight or normal weight (35.8 %). Moderate-heavy drinkers (15–21 units/week) had higher rates of overweight (47.0 %), household income in the highest quartile (quartile 4) (46.3 %), vocational education (46.9 %), and heart failure (78.0 %). Heavy drinkers (>21 units/week) had a higher proportion of smoking (28.2 %) and short or medium-length higher education levels (20.1 %). In the lowest income quartile (quartile 1), there was a higher proportion of abstainers (25.6 %), while in the highest income quartile (quartile 4) there was a higher proportion of moderate-heavy drinkers. The majority of those with basic school as the highest educational level consisted of abstainers, while those with long higher education as the highest educational level were more likely to be moderate-heavy drinkers (8.3 %) (Table 1).

**Fig. 2:**
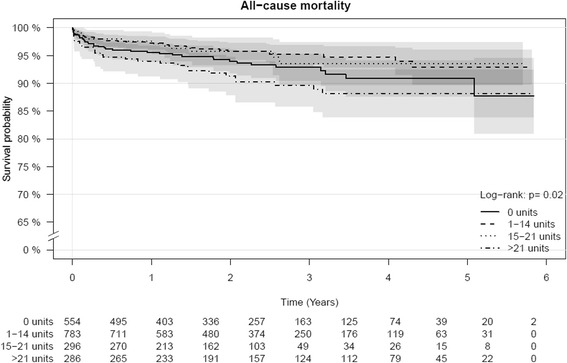
Kaplan-Meier survival estimates for all-cause mortality stratified by weekly alcohol consumption

Figure 2 shows the Kaplan-Meier survival estimates for all-cause mortality stratified by weekly alcohol consumption. A total of 112 (5.84 %) patients died from all-cause mortality. The curves show the different mortality rates for the four alcohol groups (log-rank *p*-value = 0.02).

### All-cause mortality

**Fig. 3:**
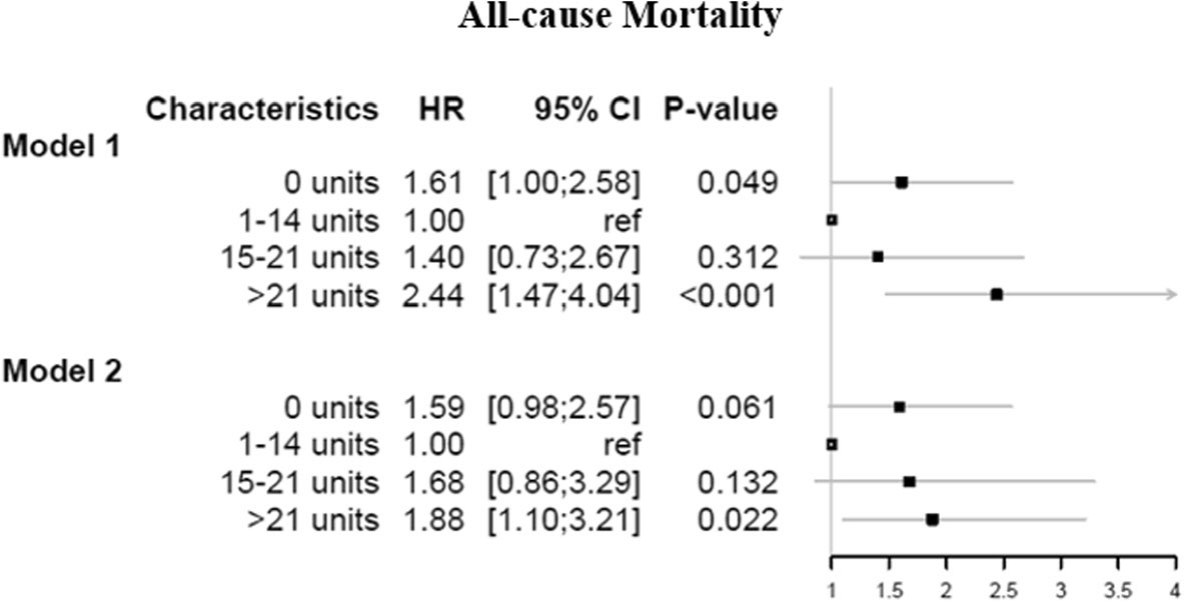
Hazard ratios and 95 % confidence intervals for all-cause mortality, as calculated by Cox regression models on the imputed data. Model 1: Adjusted for age and sex. Model 2: Adjusted for age, sex, smoking status, body mass index, educational level, household income, coronary artery bypass graft after 2008 (time), chronic obstructive pulmonary disease, acute myocardial infarction, atrial fibrillation, hypertension, liver disease, stroke, diabetes mellitus, heart failure and renal failure

The most frequent cause of death was from a cardiovascular cause (96 patients, 86 % of all deaths). Other causes of death (in all cases *n* < = 3) were sepsis, pneumonia, COPD, diverticular disease with perforation and abscess, unknown cause of mortality, traffic accident, and malignant neoplasm: colon, prostate, bladder and primary site unspecified. The risk of all-cause mortality, according to alcohol consumption in model 1 (adjusted for age and sex), was not increased for the abstainers (HR 1.61, 95 % CI, 1.00–2.58) and moderate-heavy drinkers (HR 1.40, 95 % CI, 0.73–2.67) compared with the moderate consumers. However, the heavy drinkers had a high risk of all-cause mortality compared with the moderate consumers (HR 2.44, 95 % CI, 1.47–4.04) (Fig. 3, model 1). Likewise, in the fully adjusted model 2, the abstainers and moderate-heavy drinkers did not have an increased risk of all-cause mortality (HR 1.59, 95 % CI, 0.98–2.57) and (HR 1.68, 95 % CI, 0.86–3.29), respectively, while the heavy drinkers remained highly associated with increased all-cause mortality rate (HR 1.88, 95 % CI, 1.10–3.21) (Fig. 3, model 2).

### Cardiovascular mortality

**Fig. 4:**
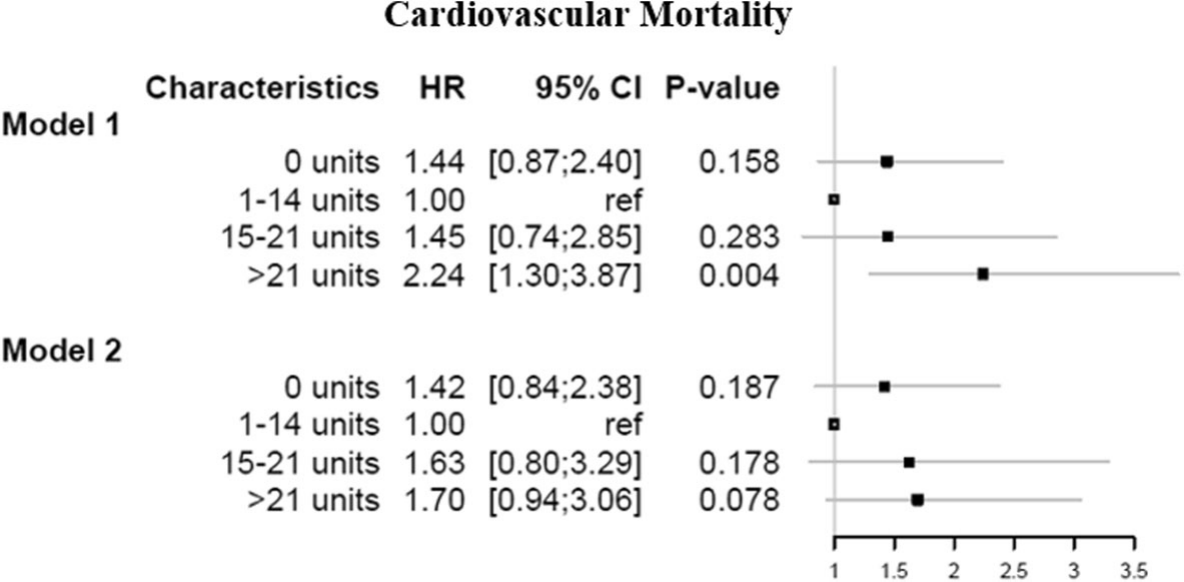
Hazard ratios and 95 % confidence intervals for cardiovascular mortality, as calculated by Cox regression models on the imputed data. Model 1: Adjusted for age and sex. Model 2: Adjusted for age, sex, smoking status, body mass index, educational level, household income, coronary artery bypass graft after 2008 (time), chronic obstructive pulmonary disease, acute myocardial infarction, atrial fibrillation, hypertension, liver disease, stroke, diabetes mellitus, heart failure and renal failure

The risk of cardiovascular mortality according to alcohol consumption in model 1 (adjusted for age and sex) was not increased for the abstainers (HR 1.44, 95 % CI, 0.87–2.40) and moderate-heavy drinkers (HR 1.45, 95 % CI, 0.74–2.85). However, the heavy drinkers had a high risk of cardiovascular mortality compared with the moderate consumers (HR 2.24, 95 % CI, 1.30–3.87) (Fig. 4, model 1). In the fully adjusted model 2, there was no increased risk of cardiovascular mortality for the abstainers (HR 1.42, 95 % CI, 0.84–2.38), moderate-heavy drinkers (HR 1.63, 95 % CI, 0.80–3.29) and heavy drinkers (HR 1.70, 95 % CI, 0.94–3.06) (Fig. 4, model 2).

### 30-day mortality

Twenty-two patients died within 30 days. In a fully adjusted model, the hazard ratio for 30-day mortality was 0.74 (95 % CI, 0.23–2.32) for the abstainers, 0.36 (95 % CI, 0.07–1.93) for the moderate-heavy drinkers and 2.20 (95 % CI, 0.65–7.36) for the heavy drinkers.

### Other analyses

Complete case analyses were performed for comparisons with primary analyses on imputed data. No increased risk was observed in model 1 for all-cause mortality among the abstainers (HR 1.57, 95 % CI, 0.95–2.57) and moderate-heavy drinkers (HR 1.32, 95 % CI, 0.66–2.65). The heavy drinkers had a high risk of all-cause mortality compared with the moderate consumers (HR 2.42, 95 % CI, 1.40–4.20). The fully adjusted model showed a risk of all-cause mortality for the abstainers (HR 1.69, CI, 1.02–2.81) and heavy drinkers (HR 2.18, CI, 1.23–3.88) but not for the moderate-heavy drinkers (HR 1.63, CI, 0.78–3.36).

In model 1, no increased risk of cardiovascular mortality was observed for the abstainers (HR 1.48, 95 % CI, 0.87–2.52) and moderate-heavy drinkers (HR 1.35, 95 % CI, 0.65–2.81). The heavy drinkers had a high risk of cardiovascular mortality (HR 2.22, 95 % CI, 1.22–4.02) compared with the moderate consumers. The fully adjusted model showed no risk of cardiovascular mortality for the abstainers (HR 1.60, 95 % CI, 0.93–2.75) and moderate-heavy drinkers (HR 1.54, 95 % CI, 0.71–3.31), while the heavy drinkers had an increased risk (HR 1.98, 95 % CI, 1.05–3.73).

This study had missing alcohol consumption data for 1,622 patients (45.8 %). Of these 1,622 patients, 466 underwent acute CABG. The *x* ^2^ tests showed no difference between the population with complete data and the group of missing alcohol consumption on education (*p*-value 0.57), diabetes mellitus (*p*-value 0.12), stroke (*p*-value 0.40), renal failure (*p*-value 0.11), liver disease (*p*-value 0.08), COPD (*p*-value 0.53) and AF (*p*-value 0.24) data.

## Discussion

This study examined the association between alcohol consumption and mortality among CABG patients. The primary result was that among CABG patients, only heavy drinkers (>21 units/week) were significantly associated with an increased risk of all-cause mortality. The risk of dying did not differ between the abstainers and moderate consumers.

### Interpretation

Our results are comparable with those of previous studies that investigated the influence of alcohol consumption on composite outcomes, including mortality, among CABG patients [14, 15]. Likewise, these studies showed no risk of increased mortality with moderate alcohol consumption. In addition, our results aligned with those from other studies, that did not target CABG patients; in these studies, moderate alcohol consumption did not adversely affect all-cause mortality and cardiovascular mortality [2–8].

In contrast to comparable studies [14, 15], our results showed an increased risk of all-cause mortality for heavy drinkers. One of these studies was not able to distinguish between heavy drinking and moderate alcohol consumption, and the other study used a lower threshold to define the highest alcohol use (≥14 units/week) than our study, thus limiting knowledge concerning heavy drinkers (>21 units/week). The different definition of heavy alcohol consumption and the lack of examining heavy drinkers might explain why our results reveal an increased risk for all-cause mortality for this specific patient group. The results of an increased risk of all-cause mortality for heavy drinkers aligned with the results of other studies not targeting CABG patients [2, 3]. Thus, our study extends this observation to include CABG patients who, consequently, do not seem to differ from the general population in terms of alcohol consumption and mortality, even though the burden of co-morbidities for CABG patients has increased over time [12, 13].

The fully adjusted model examining the association of alcohol consumption and cardiovascular mortality showed an increased cardiovascular mortality rate among heavy drinkers, but this result was not statistically significant. As shown in the forest plots (Figs. 3 and 4), the results from model 1 and model 2 for all-cause mortality and cardiovascular mortality illustrate J-shaped curves, which indicate higher mortality rates for the heavy drinkers, as reported elsewhere [3, 7, 32, 42]; thus, these results indicate an increased mortality rate among heavy drinkers. The pattern for all-cause mortality and cardiovascular mortality is nearly identical, as 86 % of all deaths are ascribed to cardiovascular causes. This majority of deaths attributable to cardiovascular mortality calls for separate investigation of this specific cause of death. However, as alcohol consumption may be related to other not-cardiovascular related causes of death (e.g. cancer), all-cause mortality is the most important outcome and the cardiovascular mortality results should be interpreted with caution. Conclusively, this result does not alter the finding that alcohol consumption seems to have little influence on mortality among CABG patients, except for heavy drinkers.

Other studies have found beneficial effects of moderate alcohol consumption on all-cause mortality and cardiovascular mortality [2–8]. The beneficial effect of alcohol is generally ascribed to increased plasma high-density lipoprotein (HDL) [43] and antithrombotic effects [44], where increased HDL levels are best established [30]. No beneficial effects of moderate alcohol consumption were observed in our study. For the sensitivity analysis, analyses were also conducted on the population with complete data. Conclusively, no major differences were observed between the analyses on the imputed data and the population with complete data; however, the risk estimates were generally slightly attenuated. Our results showed no harm for moderate-heavy drinkers regardless of whether the analysis was performed as complete cases or with multivariate imputation, thereby indicating that CABG patients do not need stricter advice to abstain from alcohol consumption, other than heavy drinking.

Previously comparable studies [14, 15] have examined alcohol consumption and composite outcomes, including mortality, for CABG patients. Therefore, our results most likely reflect a fairer examination between alcohol consumption and mortality for CABG patients because we did not use composite outcomes.

### Strength and limitations

A major limitation of our study is the observational design, which does not eliminate unmeasured confounders. Residual confounding may, therefore, be present. Due to the study design, our results should only be considered as associations and not as causal relationships.

Although this study had missing data on alcohol consumption for 1622 patients, there was no difference in relation to educational level, diabetes mellitus, stroke, renal failure, liver disease, COPD and AF between the population with complete data and the group with missing data, which decreases the possibility that the missing data introduced selection bias in the study. Of the 1622 patients, 466 underwent acute CABG, which might explain the missing information on alcohol consumption (i.e., the condition of the patient may have been too severe to report the amount of weekly alcohol consumption). Multivariate imputation was performed on the missing covariates. This technique has the advantage of increasing the size of the study population and is typically more efficient than complete cases analysis [40, 41]. Analysis with complete cases only could lead to over-or underestimation of the effect sizes [41]; thus, multivariate imputation is seen as a strength. However, the multivariate imputation did only increase the study population with 155 patients with missing values on covariates, and the high number of missing data should be recognized as a limitation.

We have adjusted for a wide range of potential confounders, which have been possible due to national registers and increases the strength of the analysis. In general, the information of the robust data obtained from the registers is regarded as high quality, and it is one of the main strengths of this study [21, 22, 27, 28, 38]. The data obtained from the national registers are collected independently of this study and, therefore, decrease the possibility of both selection and information bias. The use of the registers further adds to the strength of the study because complete follow-up data are available for all CABG patients.

The amount of alcohol consumption reported tends to be underestimated, particularly among heavy drinkers [45], and the validity of the amount of moderate alcohol consumption is more stable than heavy drinking [42]. An underestimation where some heavy drinkers by mistake is classified as moderate-heavy drinkers would probably lead to a lower association between alcohol consumption and mortality for heavy drinkers. This could also lead to an elevated risk for moderate-heavy drinkers. However, no increased risk was found for this group. Thus, underestimation of alcohol consumption for moderate-heavy drinkers would probably have little impact on the results. Nevertheless, newer studies consider the measurement of alcohol consumption as valid [46, 47]. In this study, alcohol consumption was measured as a part of the anaesthesiologist anamnesis prior to the operation because alcohol consumption can impact the administration of anaesthesia. The measurement of alcohol consumption obtained in connection with the comprehensive CABG procedure is, therefore, expected to be reliable.

We were not able to identify the different types of alcohol that could have potentially influenced the results if one type of alcohol is more harmful or beneficial than another. Compared to a moderate intake of beer or liquor, moderate wine drinking has been associated with reduced mortality [48] and higher socioeconomic position [49]. We have adjusted for income and educational level as a proxy for socioeconomic position. The reduced association between moderate wine drinking and mortality may, however, be ascribed to more favourable drinking habits and socioeconomic position than wine *per se* [49]. Nevertheless, it would have been favourable to have access to separate information on wine, beer and liquor. It has been proposed that it is alcohol *per se* that has a protective effect on mortality rather than different types of alcohol [5, 30, 50]. In this study, we found no beneficial effect of moderate alcohol consumption, and the lack of separating different types of alcohol should, therefore, not affect the results crucially.

The use of all-cause mortality as an outcome had the advantage that it requires no further ascertainment than the time of death, and therefore, it will tend to eliminate the possibility of information bias for the outcomes. However, this issue could occur depending on the cardiovascular mortality because the correctness of cause-specific mortality depends on an individual physician’s registration of the death certificate, which might lead to misclassification and possible bias if the specific cause of death registration was dependent on alcohol consumption. Nevertheless, there seems to be a high validity of causes of death compared to clinical records [25].

## Conclusion

Only heavy drinking (>21 units/week) was significantly associated with increased mortality among CABG patients. The risk of dying did not differ between the abstainers and moderate consumers. Alcohol consumption seems to have little influence on mortality among CABG patients, except for heavy drinkers. Given the wide confidence intervals, other than to abstain from heavy drinking, further studies are necessary to justify advising patients regarding alcohol consumption.

## Abbreviations

AF: Atrial fibrillation
AMI: Acute myocardial infarction
ANOVA: Analyses of variance
ATC: Anatomical therapeutic chemical classification
BMI: Body mass index
CABG: Coronary artery bypass graft
CHD: Coronary heart disease
COPD: Chronic obstructive pulmonary disease
CPR-number: Personal identification number
DKK: Danish kroner
HDL: Plasma high-density lipoprotein
ICD-10: International classification of diseases, version 10
MICE: Multivariate imputation by chained equations
NOMESCO: Nordic medico-statistical committee
UNESCO: United Nations Educational, Scientific and Cultural Organization
